# Characterization the regulation of herpesvirus miRNAs from the view of human protein interaction network

**DOI:** 10.1186/1752-0509-5-93

**Published:** 2011-06-13

**Authors:** Zhenpeng Li, Fei Li, Ming Ni, Peng Li, Xiaochen Bo, Shengqi Wang

**Affiliations:** 1 Department of Biotechnology, Beijing Institute of Radiation Medicine, No.27, Taiping Road, Haidian District, Beijing 100850, China

## Abstract

**Background:**

miRNAs are a class of non-coding RNA molecules that play crucial roles in the regulation of virus-host interactions. The ever-increasing data of known viral miRNAs and human protein interaction network (PIN) has made it possible to study the targeting characteristics of viral miRNAs in the context of these networks.

**Results:**

We performed topological analysis to explore the targeting propensities of herpesvirus miRNAs from the view of human PIN and found that (1) herpesvirus miRNAs significantly target more hubs, moreover, compared with non-hubs (non-bottlenecks), hubs (bottlenecks) are targeted by much more virus miRNAs and virus types. (2) There are significant differences in the degree and betweenness centrality between common and specific targets, specifically we observed a significant positive correlation between virus types targeting these nodes and the proportion of hubs, and (3) K-core and ER analysis determined that common targets are closer to the global PIN center. Compared with random conditions, the giant connected component (GCC) and the density of the sub-network formed by common targets have significantly higher values, indicating the module characteristic of these targets.

**Conclusions:**

Herpesvirus miRNAs preferentially target hubs and bottlenecks. There are significant differences between common and specific targets. Moreover, common targets are more intensely connected and occupy the central part of the network. These results will help unravel the complex mechanism of herpesvirus-host interactions and may provide insight into the development of novel anti-herpesvirus drugs.

## Background

Herpesviruses are members of Herpesviridae family, a large family of DNA viruses that cause chronic, latent and recurrent infections in animals and humans. Herpesviruses are double-stranded DNA viruses with large genomes encoding complex virus particles and enzymes involved in a variety of cellular process, including nucleic acid metabolism, DNA synthesis, and protein processing [1]. In addition to herpesvirus proteins associated with pathogenic processes, herpesvirus-encoded microRNAs (miRNAs) have been also shown to play an indispensable role in herpesvirus pathogenesis [2]. miRNAs are a class of endogenous, single strand RNAs, approximately 22 nucleotides long that bind to 3'untranslated regions of transcript causing degradation of their respective targets or block protein translation. Since the discovery of virus-encoded miRNAs in Epstein-Barr Virus (EBV) [3], the roles of virus encoded miRNAs in the regulation of the viral life cycle and in mediating interactions between viruses and their hosts, have been examined in some detail [4].

With the emergence of versatile miRNA target prediction algorithms and availability of proteome-wide protein-protein interaction data sets, manually curated or derived from high-throughput experiments (such as a yeast two-hybrid screen), it has become possible to investigate regulation of the whole human PIN by miRNAs. Since protein-protein interactions constitute the basis of most life processes, such studies might provide important clues necessary to the thorough understanding of biological mechanisms at the whole systems level. In recent years, human miRNA regulated cellular networks, such as signal transduction, gene regulatory network, PIN and metabolic network, have been studied in great detail [5-9]. Some of the results highlight an interesting commonality: that miRNAs tend to target nodes with high topological complexity, such as hubs and bottlenecks. In signal transduction network, miRNAs preferentially target downstream network components, positively linked network motifs and downstream components of the adaptors that have the potential of recruiting additional downstream components [5]. Genes in regulatory networks with more transcription factor binding sites have, on average, more miRNA-binding sites and a higher probability of being targeted by miRNAs [6]. Protein degree in the human PIN correlates to the number of miRNA target-site types of the gene encoding the respective protein [7]. In addition, analysis of the human PIN and the human metabolic network showed that human-encoded miRNAs preferentially target hubs and bottlenecks [8,9].

miRNAs are some of the key regulators of various biological processes, for example, they play an important role in virus-host interactions [2-4]. This applies both to human-encoded and virus-encoded miRNAs. We need to examine the mechanisms involved in such interactions to gain insight into this complex process. To date, only one study has systematically examined the functional characteristics of human herpesvirus miRNAs [10]. The results of that study showed a statistically significant preferential targeting of host genes involved in cellular signalling and adhesion junction pathways. Other studies mentioned above revealed some of the regulatory characteristics of human encoded miRNAs in biological networks, however, in the field of virus miRNA-mediated virus-host interactions, not many studies have been conducted at the systems level. In this report, we explored the topological characteristics of human herpesvirus miRNAs that target human PIN. We believe that determining which human proteins are targeted by viruses will provide insight into molecular processes shared by related viruses. Taking into account the large differences between miRNAs encoded by different viruses [11], it is not unreasonable to expect that the analysis of one virus group, in our case the herpesviruses, will yield some interesting results. As essential cellular building blocks, proteins perform a variety of functions by interacting with other proteins. If we are to achieve comprehensive understanding of herpesvirus-host interactions and better understand the molecular basis of viral pathogenesis, it will be of great importance to study the function of herpesvirus miRNAs in the framework of PIN. The results of these studies are also likely to provide new means for developing novel therapeutic strategies for the treatment and prevention of viral infections.

## Results

### Herpesvirus miRNAs preferentially target hubs and bottlenecks

**Figure 1:**
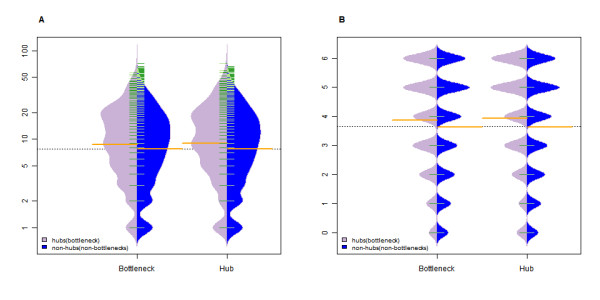
**Comparison of herpesvirus miRNAs regulation strength for hubs (bottlenecks) and non-hubs (non-bottlenecks)**. Purple areas represent the estimated density of the hub distributions (bottlenecks) and blue areas represent non-hub (non-bottlenecks) areas. (A) Comparison of the estimated density of distribution of miRNAs numbers between hubs (bottlenecks) and non-hubs (non-bottlenecks). (B) Comparison of the estimated density of the distribution of virus types numbers between hubs (bottlenecks) and non-hubs (non-bottlenecks). The green and yellow lines show the values for individual observations and their mean values, respectively.

There are two known features of human miRNA regulatory properties [7,8]: first, it's their preferential targeting of hubs and bottlenecks; second, there is a highly significant positive correlation between protein degree in human PIN and the number of target-site types at the 3'UTR region of its gene. It has been established that herpesvirus-encoded miRNAs are processed and mature within human cells, which hints that they display properties similar to those of human-encoded miRNAs. To investigate this possibility, we defined hubs and bottlenecks as 5% of PIN nodes with the highest degree and betweenness centrality and analyzed the significance of hubs and bottlenecks targeted by herpesvirus miRNAs on two levels. First, to inspect whether the targets of herpesvirus miRNAs cover more hubs than random conditions, the statistical significance of the proportion of miRNA-targeted hubs (bottlenecks) was tested. The results demonstrated that herpesvirus miRNAs preferentially target more these nodes (Table 1). Second, the nodes targeted by herpesvirus miRNAs were classified into two groups: hubs (bottlenecks) and non-hubs (non-bottlenecks). The regulation strength of the herpesvirus miRNAs in the two groups was next examined. The statistical significance of virus miRNAs and virus type numbers for the two groups was tested, with positive results for both (P value: 0.0004 (0.028) and 0.0006 (0.007); permutation test). In addition, as an alternative to the boxplot, beanplots [12] were employed to visualize the estimated density of the distribution of virus miRNAs and virus type numbers for the two groups, respectively (Figure 1). As hubs are the crucial nodes in the PIN, preferential hub targeting will make herpesvirus miRNAs more efficient in the context of that network. To further analyze the herpesvirus miRNAs targeting behaviour, we compared the nodes targeted by those miRNAs with nodes targeted by human-encoded miRNAs. We found that the two node sets behaved similarly (Table 2). Two possibilities were proposed for the result. First, as human miRNAs are key regulators of many fundamental biological processes, some of these biological processes may be needed by viruses for successful infection. Namely, this result may be archived by the adaption of virus to its host for survival over long-term of evolution, but it is not always true considering both types of miRNAs may express at the different temporal and spatial condition. Second, some features of genes have been formed during evolution to make these genes more favorable to be targets of miRNAs, such as UTR Context [13], site accessibility [14].

**Table 1:** Herpesvirus miRNAs targeting propensity for hubs and bottlenecks

Type	miRNA-targeted Hub proportions	miRNA-targeted Bottleneck proportions
miRNA-Targets	0.9309	0.9158
Random chosen nodes (mean)	0.8769	0.877
p-value	<0.0001	0.003

P-values were computed using randomization tests.

**Table 2:** The relationship between the targeting of herpesvirus miRNAs and human miRNAs

	Virus miR	Non-virus miR
Human miR	7931	203
Non-human miR	265	871

P-value < 2.2e-16, computed by using Fisher's Exact Test

### Characteristics of common and specific targets

**Figure 2:**
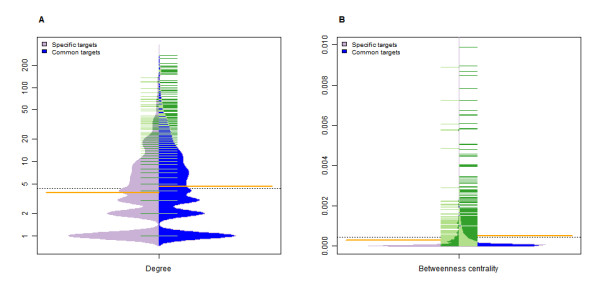
**Regulation comparison of herpesvirus miRNAs to common and specific targets**. Purple areas represent the estimated density of the distribution of specific targets and blue areas represent the estimated density of the distribution of common targets. (A) Represents the distribution of degree, and (B) the distribution of betweenness centrality. The green and yellow lines show individual observations and their mean values, respectively.

**Figure 3:**
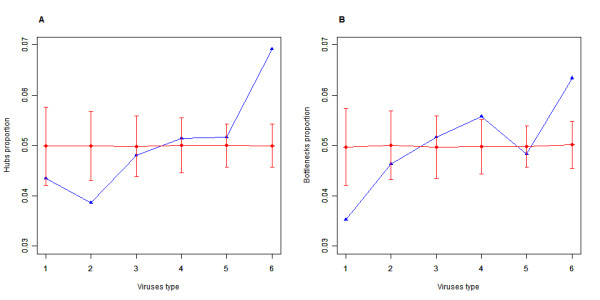
**The relationship between virus type and the proportion of hubs**. Relationship between virus type and the proportion of hubs (bottlenecks). (A) Describes hubs and (B) bottlenecks. The blue curve denotes the actual hub or bottleneck proportions for different virus types and the red curve shows the simulated hub or bottleneck proportions for the nodes randomly chosen from different virus types 1,000 times, preserving the node numbers of each virus type ± standard deviation.

We defined nodes targeted by all six viruses as common targets and nodes targeted by only one virus as specific targets. There are significant differences observed in degree and betweenness centrality between common and specific targets (p value: <0.0001 and 0.0054; permutation test). The distribution differences between these two types of targets are depicted in Figure 2. To dissect further the relationship between nodes targeted by different virus types and topological attributes of the PIN, we defined the virus types of each node by counting the virus species whose miRNAs target the node. Then, the nodes were divided into six groups based on virus types, the actual hub proportion in each group was computed (Figure 3). We found a significant positive correlation between virus types and the proportion of hubs (bottlenecks) (correlation coefficient = 0.9429 and 0.8286, one sided p value = 0.0083 and 0.0292; Spearman's test). This is consistent with the observation that common targets have significantly higher degree and betweenness centrality than that of specific targets. We performed a simulation to test whether the trends were significant, that is, we re-computed the hub proportion 1, 000 times with randomly chosen nodes (the same number of nodes as hubs) and the trends seemed to be significant. The hub proportion and the proportion of bottlenecks are significant for both common and specific targets compared to the simulation. We propose that common targets might be related to the pathogenesis processes common to all the viruses and that specific targets are involved in the infection processes specific to a particular virus type.

We also investigated the significant GO terms for the common targets (Table 3) and found that most over-represented GO terms are related to various developmental and regulatory processes, such as nervous system development and regulation of signalling pathway and cell communication, indicating a close relationship between these processes and herpesvirus pathogenesis.

**Table 3:** The GO enrichment results of common targets

GO-ID	P-value	Description
32502	8.73E-10	developmental process
7399	3.98E-09	nervous system development
7275	9.03E-09	multicellular organismal development
32501	2.04E-08	multicellular organismal process
48856	9.07E-08	anatomical structure development
19226	1.00E-07	transmission of nerve impulse
48731	2.24E-07	system development
7268	3.75E-07	synaptic transmission
35466	4.23E-06	regulation of signaling pathway
10646	1.42E-05	regulation of cell communication

### Topological characteristics of common herpesvirus miRNA targets

**Figure 4:**
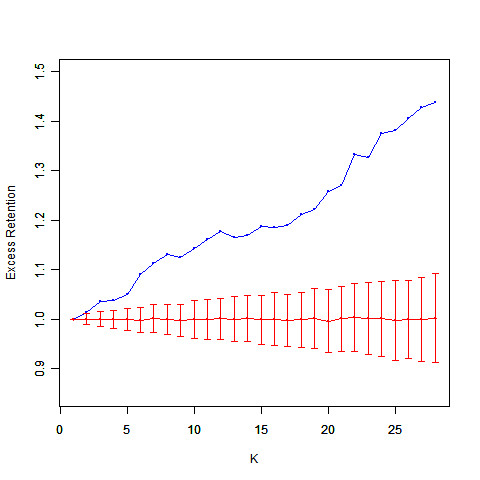
**The k-core and ER analysis of common targets**. K denotes the sub-graph with each node's degree not smaller than k. With the increment of k, higher ER values represent the central placement of the selected nodes in the original graph. The blue curve represents the actual ER. The red curve represents the simulated ER (simulation with randomly selected nodes 1,000 times preserving the number of nodes) of each k-core ± standard deviation. The comparison between the actual curve and simulated curve indicates that the trend of the actual curve is significant.

We used k-core and excess retention (ER) analysis [15] to measure the distance between common targets and the topological center. With an increasing k value, higher ER values are indicative of common targets being closer to the global center of the PIN (Figure 4). To test the significance of this observation, we randomly chose the same number of nodes as common targets and re-calculated the ER that revealed only minor fluctuations. A substantial difference in the ER for each k-core could be observed between common targets and random controls, suggesting that common targets might interconnect tightly in the PIN. To test this hypothesis, we defined strategies for measuring the significance of the module characteristics to the common targets. The first parameter was the number of nodes in the GCC comprised of common targets, the second was the density of the sub-network formed by those targets and results showed that both density and GCC are significant (Table 4). It suggested that the module formed by the common targets position in the network's core; it might be not accidental that most viruses utilized these nodes as targets. In the context of virus-host interactions, it could be beneficial for a virus to hijack the network core since this would facilitate rapid transmission of information to the rest of the network, thereby maximizing viral control of cellular functions.

**Table 4:** The modularity of sub-network formed by common targets

	Random nodes( mean)	Common targets	P-value
GCC size	948.5289	1161	<0.0001
Subnet dense	0.0001	0.0016	<0.0001

## Discussion

It is well understood that cellular functions are carried out using various specialized groups of molecules interacting via intricate networks. No approach to complex systems can succeed without exploiting network topology [16]. In this study, we investigated characteristics associated with the targeting of herpesvirus miRNAs to proteins in the human PIN. Virus-encoded miRNAs have unique advantages: they can function at the RNA level, affecting the expression of many genes rapidly and extensively. The results of this study will contribute to a better understanding of the complex herpesvirus-host interactions at the miRNA level.

We found that herpesvirus miRNAs preferentially target PIN hubs and bottlenecks, a process similar to that of human-encoded miRNAs. The biological networks displayed scale-free characteristics, *i.e*. most of the nodes have a relatively low degree, making them resistant to attacks on random nodes [17]. It seems that the vulnerability of human protein networks (only a few nodes have a high degree) is successfully exploited by herpesviruses, suggesting that these viruses must have evolved to target key nodes preferentially, allowing them to take maximum control of the human protein network during infection. Although the various roles carried out by virus-encoded proteins have been extensively studied over the past few decades, it is only recently that viral protein-regulated PIN have been studied in detail. Matthew D. Dyer *et al*. [18] examined some human-pathogen protein-protein interactions and found that both viral and bacterial pathogens interacted with human PIN hubs and bottlenecks.

The results of our comparison between common and specific targets suggested that some topological differences existed between nodes related to processes associated with common and specific virus pathogenesis mechanisms. Furthermore, the significant hub and bottleneck proportions for common targets validated the preference of viral miRNAs for hubs and bottlenecks. These results provided valuable information that will help unravel mechanisms associated with herpesvirus pathogenesis.

We also characterized the modularity of common PIN targets. We found that common targets tend to form a larger module and have a higher density than randomly chosen nodes and that they are located in the global central core of PIN. During virus-host interactions, viruses use their limited resources to exploit fundamental cellular processes for finishing their life cycles. From that perspective, targeting nodes located in the central part of PIN seems a reasonable strategy designed to affect nodes in other parts of the network efficiently and rapidly. The nodes in the central part represent fundamental components of the cell, the control over which might be necessary for the virus to infect successfully. As our GO analysis confirmed, these nodes are related to processes associated with fundamental cellular regulation and development indispensable to viral survival.

To test the robustness of our results, two additional algorithms, miRanda [19] and TargetScan [20], were used to predict the herpesvirus miRNAs targets; meanwhile, a high confidence protein-protein interaction data set, HPRD (Human Protein Reference Database)-filtered, was also used to construct the PIN. Most results are in agreement with those obtained using PITA algorithm [14] and HPRD dataset [21]. The detailed results are described in additional files (see additional files 1, 2 and 3).

In this paper, we focused on the description of statistically significant, functional characteristics of herpesvirus miRNAs involved in the process of regulation of the human PIN. Some limitations to the analysis described in this report are, first, the PIN used was incomplete and therefore subject to considerable error rates; second, the large number of predicted miRNA targets makes experimental validation rather difficult. Moreover, we know that the results of different types of predictions do not fully agree with each other [13]. Our predicted herpesvirus miRNAs collection might not be complete; that is, the use of improved miRNA prediction algorithms and a wider implementation of high-throughput techniques might identify new miRNAs. Third, the herpesvirus miRNA mediated human protein interaction network, in the context of herpesvirus protein and human protein interactions, was not analyzed due to the disequilibrium and lack of herpesvirus protein and human protein interaction data. Despite these limitations, our analysis of herpesvirus miRNA interactions with the human PIN should help to reveal a broader picture of their functional mechanisms at the systems level and add to our knowledge of the viral pathogenesis process.

## Conclusions

In this study, we explored the ability of herpesvirus miRNAs to target the human PIN. Viral miRNAs preferentially target PIN hubs and bottlenecks, behaviour similar to that of human-encoded miRNAs. Topological comparison between specific and common targets showed that common targets have significantly higher degree and betweenness centrality. K-core and ER analysis revealed that common targets occupy the global central part of the PIN. Furthermore, a significant modularity of common targets was found. Their crucial topological position in the PIN suggested that they might play a key role in herpesvirus pathogenesis. These results add to our understanding of herpesvirus miRNAs functions, giving us new insights into the complex process of herpesvirus-host interactions and provide information that can be used in the development of novel antiviral drugs.

## Methods

### Source of miRNAs

miRNAs sequences from six herpesviruses (HSV1, HSV2, EBV, KSHV, HCMV and BV) were downloaded from miRBase [22], including 86 precursor sequences. 138 mature sequences of miRNAs were used to predict the targets.

### miRNA target prediction

We used three miRNA prediction tools to identify miRNAs targets: PITA [14], miRanda [19] and TargetScan [20]. Using PITA, we followed standard seed parameter settings and took seeds 6-8 bases long, beginning at position 2 of the miRNA. No mismatches or loops were allowed but a single G:U wobble was allowed in 7- or 8-mers. We parsed all 3'UTRs from the reference sequences of human mRNAs that were downloaded from NCBI.

miRanda (version 3.3a) was used with following parameters: score cutoff = 140, energy cutoff ≤ -7.0, gap opening: -9.0, gap extension -4.0, 5' scaling: 4.

TargetScan (version 5.0) was also used without considering the conservation of genes and the sites with high context score percentiles (between 50 and 100) were chosen.

### Protein interaction data

HPRD, Release 9 [21], with 9,673 nodes and 39,204 protein-protein interactions (PPIs), was used to analyze the targeting propensity of virus-encoded miRNAs. Among the exclusively, experimentally derived protein-protein interaction databases, HPRD is the most complete and overlaps well with other PPI databases [23] suggesting that it is most likely to represent the full panorama of human PIN.

To obtain a high confidence data set, we filtered HPRD data by choosing the interactions supported by at least two experimental conditions or two papers resulting in the identification of 6,101 proteins and 14,583 interactions contained in the 'HPRD-filtered' set. 'HPRD-filtered' data was also used to test the robustness of the results.

We obtained the GCC (HPRD: 9,270 nodes and 38,855 interactions and HPRD-filtered: 5,527 nodes and 14,158 interactions) by removing small clusters and single nodes. All topological parameters were computed using GCC.

### Topological parameter definitions and computations

Degree denotes the number of edges linked to the specified node in the network.

The betweenness centrality *C_b_*(*n*) of node n is defined as follows [24]:

where s and t are nodes in the PIN different from n, *σ_st _*specifies the number of shortest paths from s to t, while *σ_st _*(n) denotes the number of shortest paths from s to t that lie on n. Betweenness centrality was normalized by the number of node pairs excluding n, so the value of betweenness centrality for each node is defined from 0 to 1. In the PIN, the proteins bridging two functional modules can gain higher values than within the module.

The network was displayed using Cytoscape [25] and the topological parameters were computed using the Cytoscape plugin NetworkAnalyzer [24].

### Randomization tests [26]

To test if herpesvirus miRNAs had targeting propensity for hubs, we randomly chose a group of nodes (the same number of nodes targeted by the herpesvirus miRNAs) and computed its miRNA-targeted hub proportion. We repeated this procedure 10,000 times. We defined the p-value using the fraction of the number of miRNA-targeted hub proportions under random conditions that was greater than the actual miRNA-targeted hub proportion.

We tested the statistical significance of GCC and of the density of the sub-network formed by multiple targets by randomly choosing the same number of nodes as common targets, and recomputed the GCC and the density of sub-network. We defined the p value using the fraction of GCC node numbers (GCC density) under random conditions, which was greater than the actual GCC fraction (GCC density).

### Permutation tests

Permutation tests were used to examine the significance of the herpesvirus miRNAs regulation strength for hubs and non-hubs. We started with the difference (Ds) between mean miRNAs number (virus type) of hubs and non-hubs and then shuffled the number of miRNAs (virus type) between all nodes and re-computed the difference (Dr) between hubs and non-hubs. We repeated this procedure 10,000 times and defined the p-value by the ratio of the number of Dr under random conditions, which was greater than the actual value. Similar procedures were used to test the significance of the herpesvirus miRNAs regulation strength differences between bottlenecks and non-bottlenecks and the degree of difference between common targets and specific targets.

### K-core analysis of the protein interaction network [15]

The k-core of a graph is defined as the maximum sub-graph obtained by pruning all nodes with a degree lower than k; series of k-cores are obtained by increasing the k value. The excess retention of nodes with property A in the k-core is defined by two steps: (1) computing the proportion of nodes with property A in the whole network (E^A ^= *N^A^*/*N*) and in the k-core  and (2) the excess retention (ER) obtained as . ER can be used to measure the distance of a group of nodes to the global center of the network. Aided by k-core, nodes are classified by considering both their degree and placement in the network. By recursively removing nodes with degrees less than the k, network layers can be systematically investigated. Combined with ER analysis, this procedure reveals the enrichment extent of a group of nodes in the k-core sub-graph and gives hints for their functional importance.

### GO enrichment analysis

Cytoscape plugin BiNGO (version 2.42) [27] was used to perform GO enrichment analysis. We selected the nodes in the GCC of HPRD as a reference set and chose 0.01 as significance level. Moreover, hypergeometric tests were used for statistical analysis and the Benjamini and Hochberg False Discovery Rate (FDR) procedure was used for the multiple testing correction.

## Authors' contributions

ZPL participated in the design of the study, carried out the computations and drafted the manuscript. FL, MN and PL participated in discussion and helped to draft the manuscript. SQW and XCB took part in the design of the study, drafted the manuscript and supervised the project. All authors read and approved the final manuscript.

## Supplementary Material

Additional File 1 **Robustness test using miRanda as miRNA targets prediction algorithm**. This file contains the analysis performed using miRanda predicted target set. Click here for file

Additional File 2 **Robustness test using TargetScan as miRNA targets prediction algorithm**. This file contains the analysis performed using TargetScan predicted target set. Click here for file

Additional File 3 **Robustness test using HPRD-filtered data to construct the PIN**. This file contains the analysis performed using HPRD-filtered data to construct the PIN. Click here for file
