# Supplementary material for: Characterization the regulation of herpesvirus miRNAs from the view of human protein interaction network
Source: BMC Syst Biol. 2011 Jun 13;5:93. doi: 10.1186/1752-0509-5-93 (PMC3125315; doi:10.1186/1752-0509-5-93)
Supplement: Additional File 1 — Robustness test using miRanda as miRNA targets prediction algorithm. This file contains the analysis performed using miRanda predicted target set. [file 1752-0509-5-93-S1.PDF]

Additional file 1

The results were computed by using miRanda as miRNA target prediction algorithm

Figures

Figure 1 - The comparison of herpesvirus miRNAs' regulation strength for hubs and non-hubs or bottlenecks and non- bottlenecks

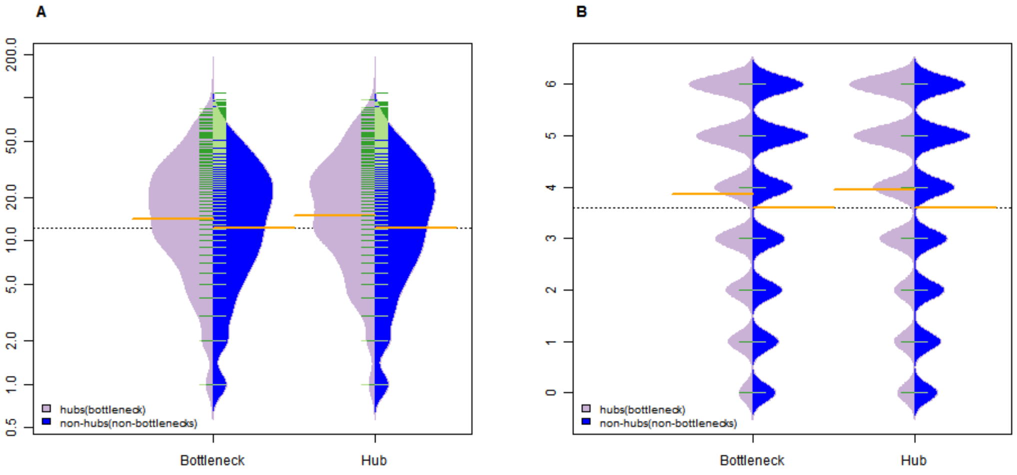

Purple areas represent the estimated density of the hub distributions (bottlenecks) and blue areas represent non-hub (non-bottlenecks) areas. (A) Comparison of the estimated density of distribution of miRNAs numbers between hubs (bottlenecks) and non-hubs (non-bottlenecks). (B) Comparison of the estimated density of the distribution of virus types between hubs (bottlenecks) and non-hubs (non-bottlenecks). The green and yellow lines show the values for individual observations and their mean values, respectively. The p values are 0.0321, 0.0001, 0.0021 and 0.0001.

Figure 2 - The comparison of herpesvirus miRNAs regulation for common and specific targets

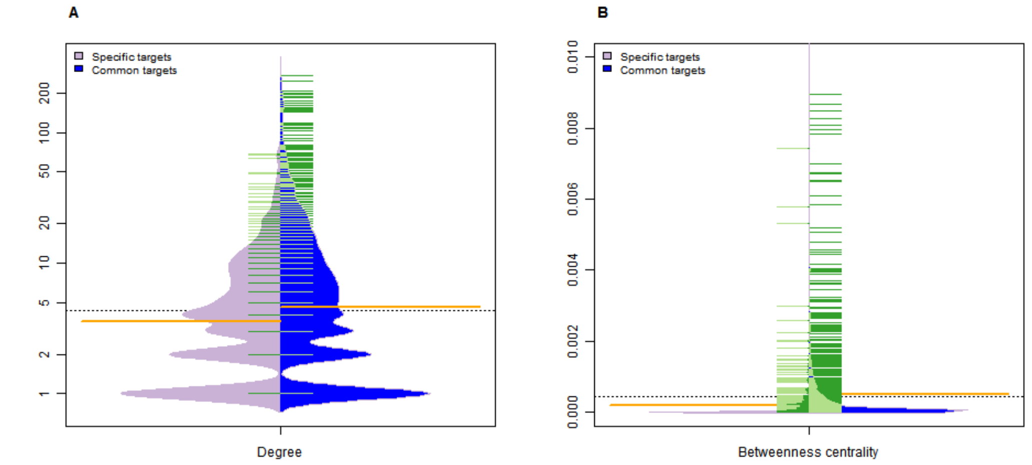

Green and yellow lines represent individual observations and their mean values, respectively. Purple areas represent the estimated density of the distribution of specific targets and blue areas represent the estimated density of the distribution of common targets. (A) Represents the distribution of degree, and (B) the distribution of betweenness centrality. The green and yellow lines show individual observations and their mean values, respectively. The p values are <0.0001 and <0.0001, respectively.

Figure 3 - The relationship between virus types and the proportion of hubs

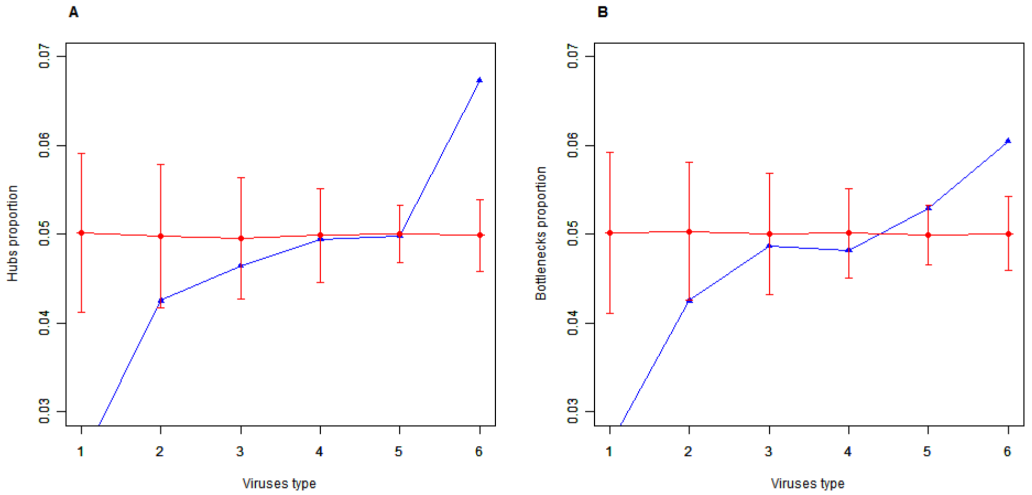

Relationship between virus type and the proportion of hubs (bottlenecks). (A) Describes hubs and (B) bottlenecks. The blue curve denotes the actual hub or bottleneck proportions for different virus types and the red curve shows the simulated hub or bottleneck proportions for the nodes randomly chosen from different virus types 1,000 times, preserving the node numbers of each virus type ± standard deviation. The correlation coefficient are 1 and 0.9429, one sided p value are 0.0014 and 0.0083 (Spearman's test).

Fig 4 - The k-core and ER analysis of common targets

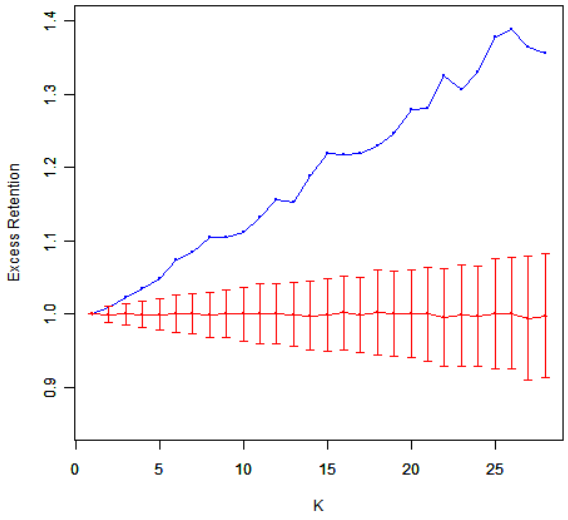

K denotes the sub-graph with each node's degree not smaller than k. With the increment of k, higher ER values represent the central placement of the selected nodes in the original graph. The blue curve represents the actual ER. The red curve represents the simulated ER (simulation with randomly selected nodes 1,000 times preserving the number of nodes of each k-core ± standard deviation. The comparison between the actual curve and simulated curve indicates that the trend of the actual curve is significant.

Tables

Table 1 - The herpesvirus miRNAs targeting propensity for hubs and bottlenecks

Type	miRNA-targeted Hubs proportion	miRNA-targeted Bottlenecks proportion
miRNA-targets	0.9460	0.9374
randomly chosen nodes (mean)	0.9188	0.9187
p-value	0.0081	0.0533

P-values were computed by randomization tests.

Table 2 - The relationship between herpesvirus miRNAs and human miRNAs targeting

	Virus miRNA	Non-virus miRNA
Human miRNA	7939	195
Non-human miRNA	579	557

The values in the table indicate the number of nodes targeted by miRNAs. P-value < 2.2e-16, computed using Fisher's Exact Test

Table 3 - The modularity of sub-network formed by common targets

	Random nodes(mean)	Common targets	P-value
GCC size	1069.0447	1278	<0.0001
Subnet density	0.0001	0.0016	<0.0001

The P-values were computed using randomization test
